# A highly efficient CMOS nanoplasmonic crystal enhanced slow-wave thermal emitter improves infrared gas-sensing devices

**DOI:** 10.1038/srep17451

**Published:** 2015-12-07

**Authors:** Andreas Pusch, Andrea De Luca, Sang S. Oh, Sebastian Wuestner, Tyler Roschuk, Yiguo Chen, Sophie Boual, Zeeshan Ali, Chris C. Phillips, Minghui Hong, Stefan A. Maier, Florin Udrea, Richard H. Hopper, Ortwin Hess

**Affiliations:** 1 The Blackett Laboratory, Department of Physics, Imperial College London, London SW7 2AZ, UK; 2 Department of Engineering, University of Cambridge, Cambridge CB3 0FA, UK; 3 Department of Electrical and Computer Engineering, National University of Singapore, 117576 Singapore; 4 Cambridge CMOS Sensors Ltd., Cambridge CB4 0DL, UK

## Abstract

The application of plasmonics to thermal emitters is generally assisted by absorptive losses in the metal because Kirchhoff’s law prescribes that only good absorbers make good thermal emitters. Based on a designed plasmonic crystal and exploiting a slow-wave lattice resonance and spontaneous thermal plasmon emission, we engineer a tungsten-based thermal emitter, fabricated in an industrial CMOS process, and demonstrate its markedly improved practical use in a prototype non-dispersive infrared (NDIR) gas-sensing device. We show that the emission intensity of the thermal emitter at the CO_2_ absorption wavelength is enhanced almost 4-fold compared to a standard non-plasmonic emitter, which enables a proportionate increase in the signal-to-noise ratio of the CO_2_ gas sensor.

The field of plasmonics^1^ has demonstrated great potential to facilitate many sensing applications^2^ such as the sensing of bio-molecules brought in contact with or in close proximity to plasmonic nano-structures through surface enhanced Raman spectroscopy^3^ or resonance shifts of plasmonic nano-particles^4^,^5^. In these local sensing schemes, Ohmic losses in the metal have been a challenge^6^,^7^, but nano-scale sources of plasmons^8^,^9^ or light^9^,^10^ are offering a remedy with improved sensitivity, as demonstrated for the detection of explosives through changes in the lasing behaviour of a lasing plasmon nanocavity^11^. Concurrently, even more established (‘remote’) sensing techniques such as non-dispersive infrared (NDIR) spectroscopy^12^, can also benefit from plasmonics on the nanoscale, particularly through enhanced absorption^13^,^14^,^15^ or adjusted thermal emission^16^,^17^,^18^. Here, we demonstrate a close to 400% increase in the sensitivity and signal-to-noise ratio of a conventional NDIR carbon-dioxide (CO_2_) gas-sensing device by replacing its standard thermal emitter with a tungsten^19^ based CMOS nanoplasmonic crystal thermal emitter that exploits a slow-wave plasmonic lattice resonance together with controlled spontaneous thermal plasmon generation and elevated plasmon-to-light coupling to achieve a 4-fold enhancement of infrared light emission.

NDIR spectroscopy provides excellent stability, selectivity and sensitivity^20^ and is therefore the preferred gas sensing method widely used in e.g. air quality monitoring, medicine and the manufacturing industry. Among the gases with absorption fingerprints in the 2 μm–14 μm region of the infrared (IR) spectrum, carbon dioxide is the most commonly targeted^21^ but other gases including carbon monoxide, methane^22^, ethanol and other volatile organic compounds (VOC)^23^ are also of prime interest. NDIR sensors (see Fig. 1a) rely on the specific radiation absorption properties of each gas molecule to quantitatively estimate the target gas concentration. Typically, NDIR sensors use broadband IR sources such as filament bulbs. However, these devices are often quite bulky and suffer from high power consumption, slow transient response and aging effects. In recent years, micro-electro-mechanical systems (MEMS) based miniaturized IR thermal emitters have become available^24^,^25^,^26^. They comprise a resistive heating element embedded within a thermally isolating structure, which enables low power consumption and fast heating/cooling times^27^ and it is possible to use standard CMOS-compatible fabrication processes to fabricate MEMS based thermal emitters with good reproducibility, in volume and at low cost^28^. However, since, fundamentally, thermal emission from a structure is proportional to its emissivity, namely 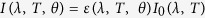, where *I*_0_(*λ*, *T*) is the blackbody spectrum, and, according to Kirchhoff’s law, the emissivity *ε*(*λ*, *T*, *θ*) of any body is equal to its absorptivity *α*(*λ*, *T*, *θ*) at the same wavelength *λ*, temperature *T* and emission angle *θ*, only a good absorber is a good thermal emitter. Unfortunately, typical CMOS materials such as semiconductors and dielectrics have low absorption coefficients in the wavelength range commonly used for NDIR gas sensing between 3 and 5 μm^27^ and current commercial thermal emitters in this range are not yet close to the theoretical efficiency limit. Metal structures, in contrast, are generally good absorbers of visible and infrared light. However, a flat sheet of metal such as, in particular, a tungsten heating element in a thermal emitter, is a good reflector for a broad range of frequencies rather than an absorber. We therefore apply nano-plasmonics and metamaterials principles to design a good plasmonic crystal IR absorber and, therefore, by Kirchhoff’s law, a good thermal emitter structure (Fig. 1b). The structure comprises a periodic array of metal (tungsten)^29^ elements, added above a micro-heater and embedded in a silicon dioxide membrane (see Fig. 1c) and meets the requirements for CMOS compatibility and high temperature stability^18^ that are paramount for low-cost fabrication of practically relevant thermal emitter devices.

As basic design for the plasmonic crystal emitter structure we employ a periodic hexagonal arrangement of tungsten (W) cylinders as shown schematically in Fig. 1c. This enables a coupling between light and surface plasmons (SPs) and these surface plasmons experience absorption due to collision processes with electrons and phonons (Fig. 1d). Because of the fundamental reciprocity of quantum electrodynamics, the process of spontaneous generation of plasmons is also possible and in thermodynamic equilibrium the emission and absorption of SPs is balanced. In the plasmonic crystal thermal emitter realised here, the SPs are spontaneously emitted on the metal surfaces and, most importantly, out-coupled efficiently to the external light field (compare: Fig. 1d,e). The frequency and angle dependence of the absorption strength of the plasmonic structure determines the frequency and angle dependence of the emitted IR light from the heated device.

For NDIR gas sensing, it is advantageous to use an IR source which is an efficient emitter at the gas absorption wavelength of interest. Broadband IR sources such as filament bulbs generate significant amounts of heat and out of band radiation which has to be heavily attenuated through the use of an optical band pass filter in order to make the NDIR system selective to the target gas. In our approach, we therefore aim to achieve a very efficient and narrowband thermal emitter to enhance emission at the CO_2_ absorption wavelength of 4.26 μm and we now apply metamaterials principles to engineer a CMOS fabricated plasmonic absorber that, when heated, efficiently generates and out-couples thermally generated SPs^30^. Tuning of the SP resonance was achieved by varying two main design parameters in the metal surface layer: the radius of the cylinders and the pitch (inter-cylinder distance) of their arrangement. To find the most advantageous plasmonic crystal configuration for thermal emission enhancement in the CO_2_ absorption wavelength range, we consider the reflectivity R, which translates into its absorptivity (and thereby emissivity) as *ε* = *α* = 1 − *R*. Figure 2b presents a map of the calculated emissivity in normal direction at 4.26 μm for varying pitch and cylinder radius of the plasmonic crystal. This provides us with guidance towards the optimal structure for emissivity enhancement at the targeted CO_2_ wavelength. We can identify two distinct modes that (incoming) light can couple into. The mode at higher pitch values (around 3.5 μm) is the gap surface plasmon polariton or gap plasmon mode (GPM) between the two metal layers, which propagates in the plane of the structure^31^. The other mode seen in the emissivity map is the surface lattice resonance (SLR), which arises from coupling between individual particle resonances^32^,^33^. Incoming light can couple efficiently to both modes and thereby be absorbed almost completely, which manifests itself in emissivity values very close to unity at resonance for both the GPM and SLR. The difference, however, lies in their spatial profile. In the case of the GPM the light is confined to the two metal surfaces below and above the silica region whereas at the SLR, the light is trapped close to the cylinders on the surface (compare: Fig. 2d,f).

The gap plasmon mode (GPM) can be externally accessed by light of 4.26 μm wavelength in plasmonic crystals with pitch sizes around 3.45 μm, with only a small dependence of the optimum pitch value on the cylinder radius. However, the position of the absorption maximum depends strongly on the emission angle. This dependence is described approximately by the grating equation ***k*** = ***k***_0_ + *m***G,** where ***k*** is the wave-vector of the waveguide mode, ***k***_**0**_ is the wave-vector of the incoming field and **G** is the momentum added by the grating and is clearly apparent in the angle-dependent emissivity map (Fig. 2c). A structure designed and optimised for the GPM can therefore act as a highly directional thermal emitter at a particular wavelength. However, the NDIR system requires an enhancement of emission over a wide range of angles to collect as much light at the relevant wavelength as possible with the parabolic mirror, and we now thus focus on the SLR. Indeed, the SLR behaves quite differently in comparison to the GPM. Optimal pitch and radius strongly depend on each other because both, a larger radius and a greater pitch, shift the SLR to longer wavelengths (see Fig. 2a,b). Therefore, in order to keep the SLR at a constant wavelength, an increase in pitch has to be counteracted by a reduction in radius and vice versa. The angular dependence of the SLR, depicted in Fig. 2c for the example of a pitch of 2.6 μm and a radius of 800 nm, is much weaker than for the gap mode. It stretches up to emission angles of 20°, without a big shift in the emission wavelength. For the application in a NDIR gas sensor this is beneficial for two reasons: Firstly, the enhancement over a wide range of angles is ensured by the angle-independence of the resonance. Secondly, the angle-independence is related to a slow-wave propagation along the surface of the structure. Therefore, in a device of finite dimensions, only very little light escapes to the side – its lateral propagation is substantially slowed down leading to spatial localisation and increased light-matter interaction.

Exploiting the advantages of the SLR, we have fabricated several plasmonic crystal thermal emitter structures with different radii and pitches close to the SLR optimum and tested their emission properties when heated. The emitted light was detected with a commercial thermopile with a CO_2_ filter and the results are shown in Fig. 3a. Within the limits of the experimental parameters, the optimal structure has a period of 2.6 μm and a cylinder radius of 800 nm, which lies inside the absorption resonance shown in Fig. 2b. The optimised plasmonic crystal device was tested as an emitter in a commercial NDIR system designed to measure varying concentrations of CO_2_ gas and its performance was compared to a device with a bare (non-plasmonic crystal) thermal emitter. From the detected response (Fig. 3), we can immediately see that the plasmonic crystal thermal emitter substantially enhances the detected emission. The noise of the measurement is predominantly due to the thermal noise of the detector and the amplifier circuitry and stays approximately constant. Altogether, the signal to noise ratio, and hence sensitivity of the NDIR system, is increased by nearly 400% compared to the sensitivity resulting from the employment of the bare emitter. FTIR measurements were made on devices fabricated from different wafer batches (see Supplementary Information.) and show that the infrared absorption peaks do not shift discernibly, indicating that the characteristics of the device are reproducible over several CMOS fabrication runs.

To summarise, we have engineered a highly efficient nanostructured plasmonic crystal thermal emitter, based on controlled spontaneous thermal generation of surface plasmons and elevated plasmon-to-light coupling as well as exploiting a slow-wave mode allowing, their efficient emission into free space. The plasmonic crystal thermal emitter was designed for wavelengths preferred in NDIR gas sensing of CO_2_ and fabricated in a standard and reproducible industrial-standard CMOS process. Application in a commercial NDIR sensing system was demonstrated and an improvement in sensitivity and signal-to-noise ratio of almost 400% achieved.

## Methods

### Design and fabrication of the nanoplasmonic crystal IR thermal emitter

**Figure 1:**
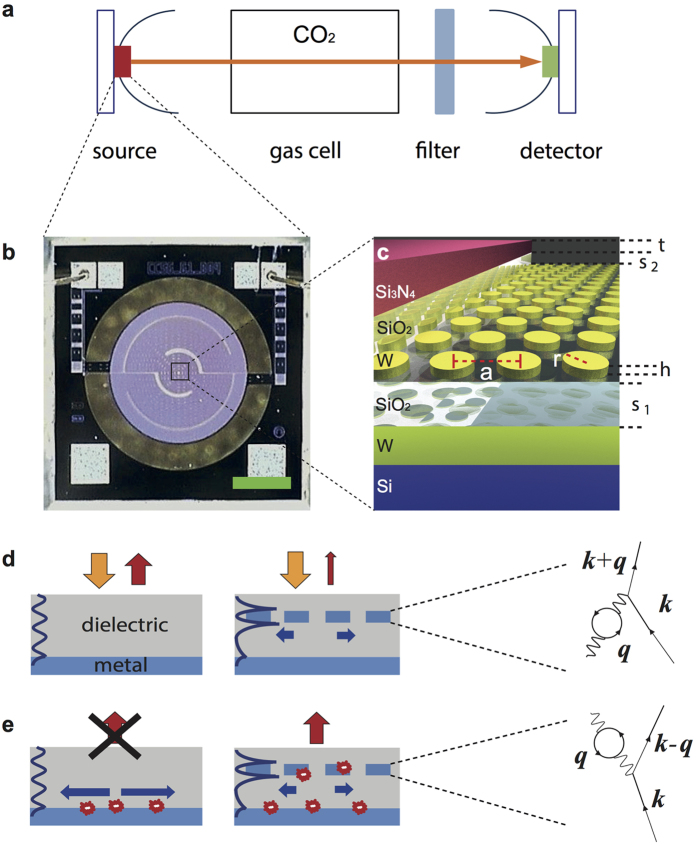
The NDIR sensing system with a plasmonic crystal slow-wave thermal emitter. (**a**) Schematic depiction of the NDIR sensing system. Light emitted from the source is collimated by a parabolic mirror, sent through a gas cell, filtered, refocused and finally detected. (**b**) A photograph of the IR emitter (green scale bar indicates 200 μm) and (**c**) a sketch of the cross-section of the device with plasmonic crystal structure (dimensions: s_1_ = 1500 nm, h = 500 nm, s_2_ = 200 nm, t = 550 nm). (**d**) Absorption: In the bare device, some of the incoming light is absorbed when it traverses the dielectric, while in the plasmonic crystal device the light is trapped in a plasmonic mode and the plasmons are subsequently absorbed in the electron gas, schematically represented by a scattering process between a surface plasmon with wave-vector q and an electron with wave-vector k. (**e**) Emission: Plasmons are spontaneously generated in the heated metal surface (the inverse process to the absorption event) but they couple to the external light-field only in the presence of a plasmonic crystal structure.

The nanoplasmonic crystal IR emitter consists of a multi-ring resistive tungsten heating element (600 μm diameter) embedded within a silicon dioxide membrane (850 μm diameter), passivated with silicon nitride. The membrane is used to thermally isolate the heater from the substrate to enhance the emitter electro-thermal efficiency. The IR emitter was fabricated using a 1.0 μm CMOS process at a commercial foundry. The CMOS process offers three metal layers, of which two were used for this design to form the plasmonic crystal and the heating element. All the metal layers are separated by silicon dioxide inter-metal dielectric layers. Tungsten, which is thermally stable and allows operation at temperatures up to 600 °C, was chosen as the metal layer. The membrane was formed by a Deep Reactive Ion Etching (DRIE) of the silicon handling substrate, with the buried silicon dioxide layer acting as effective etch stop. This results in near vertical membrane cavity sidewalls, permitting high on-wafer device packing density. The optical micro-graph of the fabricated IR emitter, showing the plasmonic structure, the heating element and the membrane is given in Fig. 1b.

### Simulation

**Figure 2:**
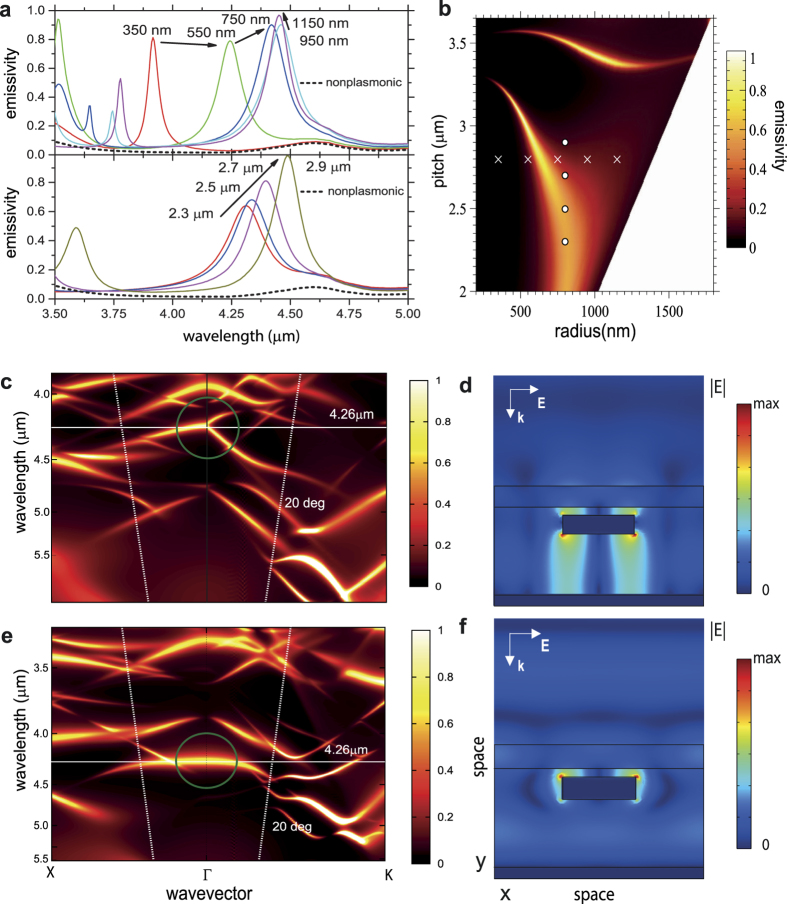
Structure-dependent emissivity. (**a**) Wavelength-dependent emissivity in the direction of the surface normal, calculated using the reflectivity R obtained from the FEM method with E = A = 1-R, for chosen pitches and radii marked with crosses and dots in the emissivity map at 4.26 μm wavelength. In the upper panel of (**a**) the pitch is kept constant at 2.8 μm and the radii are varied between 350 and 1150 nm, while in the lower panel the radius is kept constant at 800 nm and the pitch is varied between 2.3 and 2.9 μm. (**b**) shows a map of the surface normal emissivity at 4.26 μm wavelength plotted against radii and pitch of the plasmonic crystal. (**c**–**f**): Maps of the wavelength and in-plane wave-vector dependence of the emissivity for structures with (**c**) (3.4 μm pitch, 1000 nm radius) and (**e**) (2.6 μm pitch, 800 nm radius), respectively. The resonances in the green circles stems from the gap-plasmon mode and the lattice resonance respectively. The lattice resonance is almost angle-independent for small angles. Figures (**d**,**f**) show cross-sections of the field amplitudes for the gap plasmon resonance (3.4 μm, 1000 nm), with the field mostly confined in the waveguide and the lattice resonance (2.6 μm, 800 nm), with the field confined to the tungsten particles, respectively.

We used the Finite-Element Method (FEM), employing periodic boundary conditions in the plane of the device, for the calculation of the absorption at *λ* = 4.26 μm (Fig. 2a) and the absorption spectra. The transmission through the tungsten heating element is negligible, allowing us to write *α* = 1 − *R* − *T* = 1 − *R*. FEM simulations were carried out using Comsol Multiphysics. FDTD software from Lumerical was used to calculate the angle-dependent absorption/emission spectra (Fig. 2c,e). The angle variation was achieved with Bloch boundary conditions. The periodic (or Bloch) boundary conditions correspond to a device that is infinitely extended in the plane of the heater. In a finite device, e.g. a device that is not much larger than the propagation length (on the order of hundreds of micrometers) of the excited mode, some of the light can escape to the sides, an effect not captured in the numerical simulation with periodic boundary conditions. Our simulations have been performed assuming room temperature data^34^ for the complex permittivities of the constituent materials of the device. The emitter structure is operated at higher temperatures, and here, the imaginary parts of the refractive indices (extinction coefficients) are expected to rise with increasing temperature, so that emissivities increase further. We have measured temperature dependent absorptivities and find an increase in the absorptivity over the whole spectral range. Of course, the bare emitter without a plasmonic surface structure also profits from this increase.

### Measurements

Reflectance measurements (see Supplementary Information) were performed using an FTIR system (Bruker Vertex 70) combined with an IR microscopy system (Bruker Hyperion 2000, with 36 × objective and NA = 0.5) in reflection on the die. The devices with and without the nanoplasmonic crystals were packaged and integrated into a conventional CO_2_ NDIR (Non Dispersive Infrared) sensor. The NDIR sensor evaluates the concentration of carbon dioxide by monitoring the absorption of the signal in the wavelength band around 4.26 μm, the CO_2_ molecule absorption band. As shown in Fig. 1a the NDIR optical system consists of our emitter packaged with a reflector to collimate the light towards a CCS202 thermopile detector with integrated reflector and CO_2_ filter (4.26 μm, 180 nm bandwidth). A gas cell with a path length of 7.5 mm was fitted in the optical path. Emission from the structure is collected over a broad range of angles and directed through the gas cell that is alternately filled with varying concentrations of CO_2_ and purged again with dry air (see Fig. 1a). The detector consists of a thermopile detector coupled with a preamplifier. The emitters were driven using a 8 Hz sinusoidal waveform at constant amplitude (2.4 V, 72 mA max.); the detector signal was recovered using a pseudo digital lock-in technique. The sensor/gas chamber was exposed to a range of CO_2_ concentration in dry air at a constant mass flow of 150 sccm. The measurement alternated 120 s 100% dry air steps with 120 s steps of CO_2_ gas in concentration ranging from 1.5 to 100%. Detector signal and CO_2_ concentration were recorded against time.

## Additional Information

**How to cite this article**: Pusch, A. *et al.* A highly efficient CMOS nanoplasmonic crystal enhanced slow-wave thermal emitter improves infrared gas-sensing devices. *Sci. Rep.* **5**, 17451; doi: 10.1038/srep17451 (2015).

## Supplementary Material

Supplementary Information

## Figures and Tables

**Figure 3:**
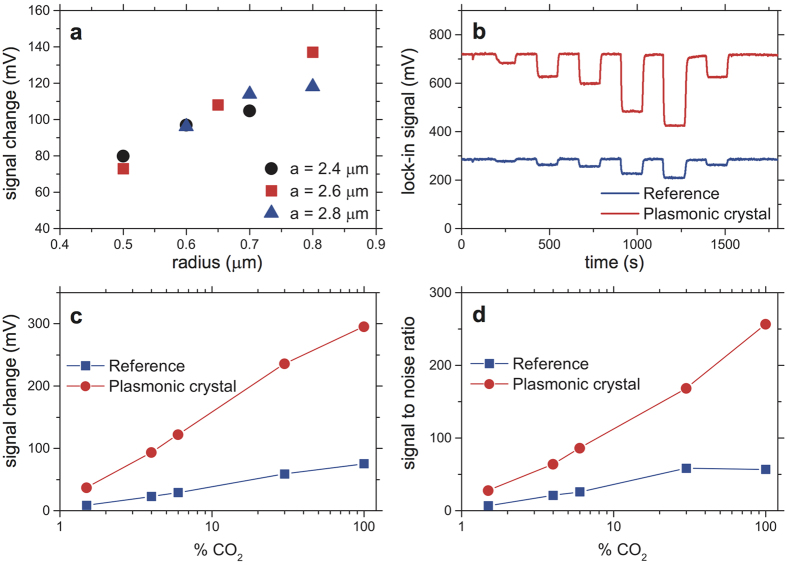
Direct emissivity measurements and sensing capabilities. (**a**) Emissivity measurements of heated samples with different radii and pitches. (**b**) Time-dependent signal obtained from NDIR measurements in a commercial gas sensing setup for the best structure from (**a**) (2.6 μm pitch, 800 nm radius) and the thermal emitter without plasmonic crystal structure. (**c**,**d**): Change in detected emission (**c**), i.e. the measured signal change, and the signal to noise ratio (**d**) in dependence on the concentration of CO_2_ for both the plasmonic crystal thermal emitter (red) and bare thermal emitter (blue). The presence of the plasmonic crystal enhances the signal to noise ratio by approximately 400%.
