# Supplementary material for: A highly efficient CMOS nanoplasmonic crystal enhanced slow-wave thermal emitter improves infrared gas-sensing devices
Source: Sci Rep. 2015 Dec 7;5:17451. doi: 10.1038/srep17451 (PMC4671012; doi:10.1038/srep17451)
Supplement: Supplementary Information [file srep17451-s1.pdf]

A Highly Efficient CMOS Plasmonic Crystal Enhanced Slow-Wave Thermal Emitter Improves Infrared Gas-Sensing Devices

Andreas Pusch, Andrea De Luca, Sang Soon Oh, Sebastian Wuestner, Tyler Roschuk, Yiguo Chen, Sophie Boual, Zeeshan Ali, Chris C. Phillips, Minghui Hong, Stefan A. Maier, Florin Udrea, Richard H. Hopper, Ortwin Hess

Supplementary Information

Methods:

Design and fabrication of the IR thermal emitter

The IR emitter consists of a multi-ring resistive tungsten heating element (600µm diameter) embedded within a silicon dioxide membrane (850µm diameter), passivated with silicon nitride. The membrane is used to thermally isolate the heater from the substrate to enhance the emitter electro-thermal efficiency.

The IR emitter was fabricated using a 1.0µm CMOS process at a commercial foundry. The CMOS process offers three metal layers, of which two were used for this design to form the plasmonic crystal and the heating element. All the metal layers are separated by silicon dioxide inter-metal dielectric layers. Tungsten, which is thermally stable and allows operation at temperatures up to 600°C, was chosen as the metal layer. The membrane was formed by a Deep Reactive Ion Etching (DRIE) of the silicon handling substrate, with the buried silicon dioxide layer acting as effective etch stop. This results in near vertical membrane cavity sidewalls, permitting high on-wafer device packing density. The optical micro-graph of the fabricated IR emitter, showing the plasmonic structure, the heating element and the membrane is given in Fig.1b.

Simulation

We used the Finite-Element Method (FEM), employing periodic boundary conditions in the plane of the device, for the calculation of the absorption at λ =4.26µm (Figure 2A) and the absorption spectra. The transmission through the tungsten heating element is negligible, allowing us to write $\alpha = 1 - R - T = 1 - R$. FEM simulations were carried out using Comsol Multiphysics. FDTD software from Lumerical was used to calculate the angle-dependent absorption/emission spectra (Figure 2C and E). The angle variation was achieved with Bloch boundary conditions.

The periodic (or Bloch) boundary conditions correspond to a device that is infinitely extended in the plane of the heater. In a finite device, e.g. a device that is not much larger than the propagation length (on the order of hundreds of micrometers) of the excited mode, some of the light can escape to the sides, an effect not captured in the numerical simulation with periodic boundary conditions. Our simulations have been performed assuming room temperature data [29] for the complex permittivities of the constituent materials of the device. The emitter structure is operated at higher temperatures, and here, the imaginary parts of the refractive indices (extinction coefficients) are expected to rise with increasing temperature, so that emissivities increase further. We have measured temperature dependent absorptivities and find an increase in the absorptivity over the whole spectral range. Of course, the bare emitter without a plasmonic surface structure also profits from this increase.

Measurements

Reflectance measurements (see Suppl. Mat.) were performed using an FTIR system (Bruker Vertex 70) combined with an IR microscopy system (Bruker Hyperion 2000, with 36× objective and NA=0.5) in reflection on the die.

The plasmonics and non plasmonics devices were packaged and integrated to a CO_2 NDIR (Non Dispersive Infrared) sensor. The NDIR sensor evaluates the concentration of carbon dioxide by monitoring the absorption of the signal in the wavelength band around 4.26µm, the CO_2 molecule absorption band. As shown in Figure 1a the NDIR optical system consisted of our emitter packaged

with a reflector to collimate the light towards a CCS202 thermopile detector with integrated reflector and CO_2 filter (4.26µm, 180nm bandwidth). A gas cell with a path length of 7.5mm was fitted in the optical path. Emission from the structure is collected over a broad range of angles and directed through the gas cell that is alternately filled with varying concentrations of CO_2 and purged again with dry air (see Figure 1A).

The detector consisted of a thermopile detector coupled with a preamplifier. The emitters were driven using a 8Hz sinusoidal at constant amplitude (2.4-1V, 72mA max.); the detector signal was recovered using a pseudo digital lock-in technique.

The sensor/gas chamber was exposed to a range of CO_2 concentration in dry air at a constant mass flow of 150 sccm. The measurement alternated 120s 100% dry air steps with 120s steps of CO_2 gas in concentration ranging from 1.5 to 100%. Detector signal and CO_2 concentration were recorded against time.

Supplementary Information:

Absorptivity from Fourier Transform Infrared Spectroscopy (FTIR)

We performed several FTIR reflection measurements on the plasmonic and non-plasmonic (bare) devices. These measurements work with focusing lenses, so that the light is incident from a large spread of angles with a numerical aperture of 0.5 and a mean angle of 20 degree. This complicates the comparison with the simulation results for normal incidence and the results obtained from the FTIR can only give an indication towards the emission performance of the devices at small angles. Also, light emitted from the side of the device or reflected at very high angles is not collected and consequently the formula 1-R=A is only approximate for this data. The purpose of these results is therefore mainly to examine some trends for the absorptivity/emissivity at different pitches and radii, and for different temperatures. We also use these measurements to verify that devices produced in different production runs have similar characteristics.

Figure S1 shows spectra for devices with different plasmonic surface structure, i.e. varying radii and pitches of the hexagonal arrangement of cylinders. We see that an increased pitch, the peak around 4 micron shifts to higher frequencies. This is analogous to the numerical results at normal incidence (see Figure 2 in the main text). The peak shift with changing radii, on the other hand, is not as pronounced as for the normal incidence simulation results. The peak at 5.5 micron (not present at normal incidence), which stems from a higher angle resonance (compare Fig 3 in the main text), is not affected by the change in the surface structure.

In Figure S2 the same arrangement of tungsten cylinders is attempted in two different production runs. We see that the peaks have not shifted, which indicates a good reliability of the production process. The absolute magnitude is slightly different, however.

Figure S3 shows temperature dependent absorptivity/emissivity results. The heated devices exhibit higher absorptivity across the whole spectral range, while the position of the peaks does not shift. This means that a device for the spectral position of its emissivity at room temperature stays spectrally optimised when heated, i.e. when in operation. The overall increase in absorptivity for high temperatures also means that even non-optimal devices can still show good emissivity when they are operated. Note that this increase in absorptivity, and therefore emissivity, adds to the usual increase of emission with temperature given by Planck's law for blackbodies.

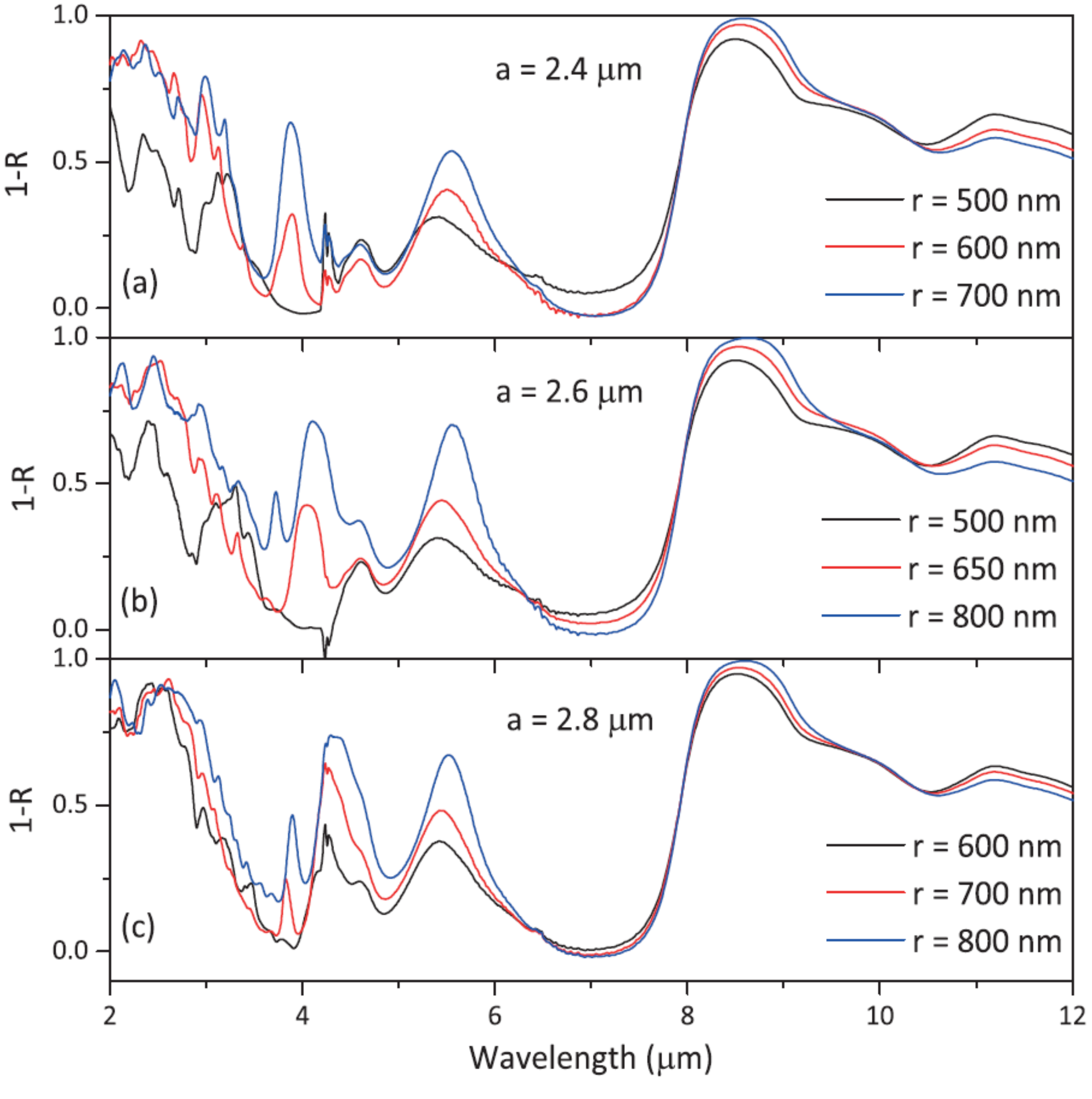

Figure S1: FTIR spectra for devices with several pitches and radii. (a) 2.4 μm (b) 2.6 μm (c) 2.8 μm.

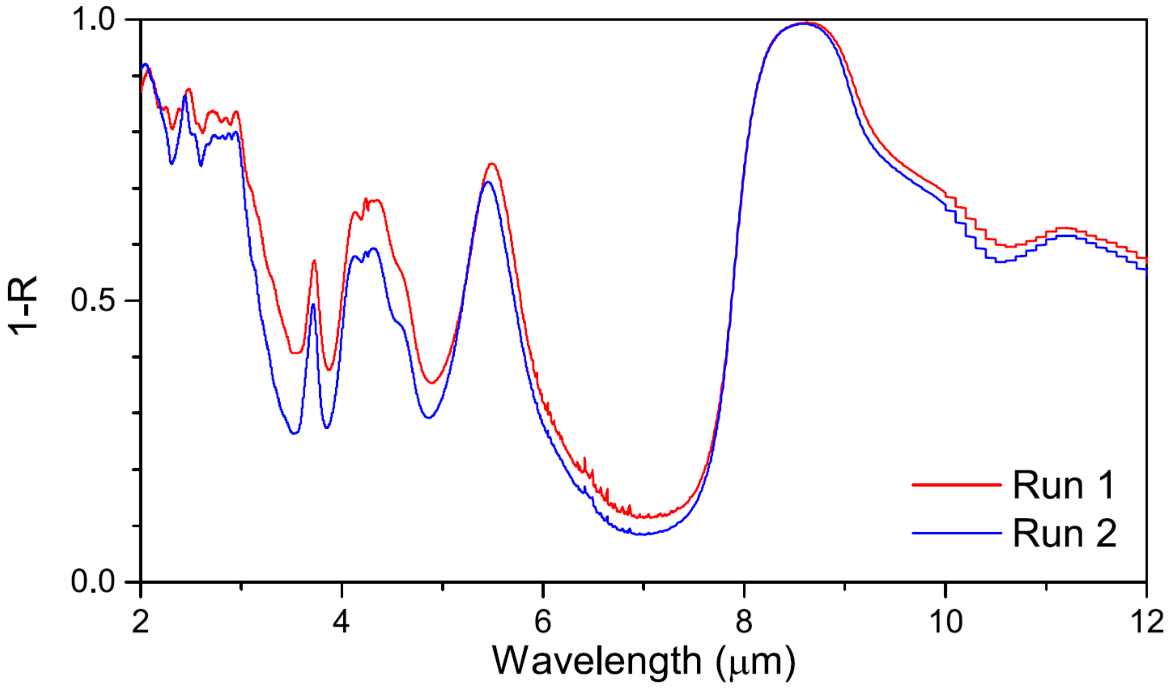

Figure S2: FTIR spectra for the optimum (2.6 μm, 0.8 μm) device in two different production runs.

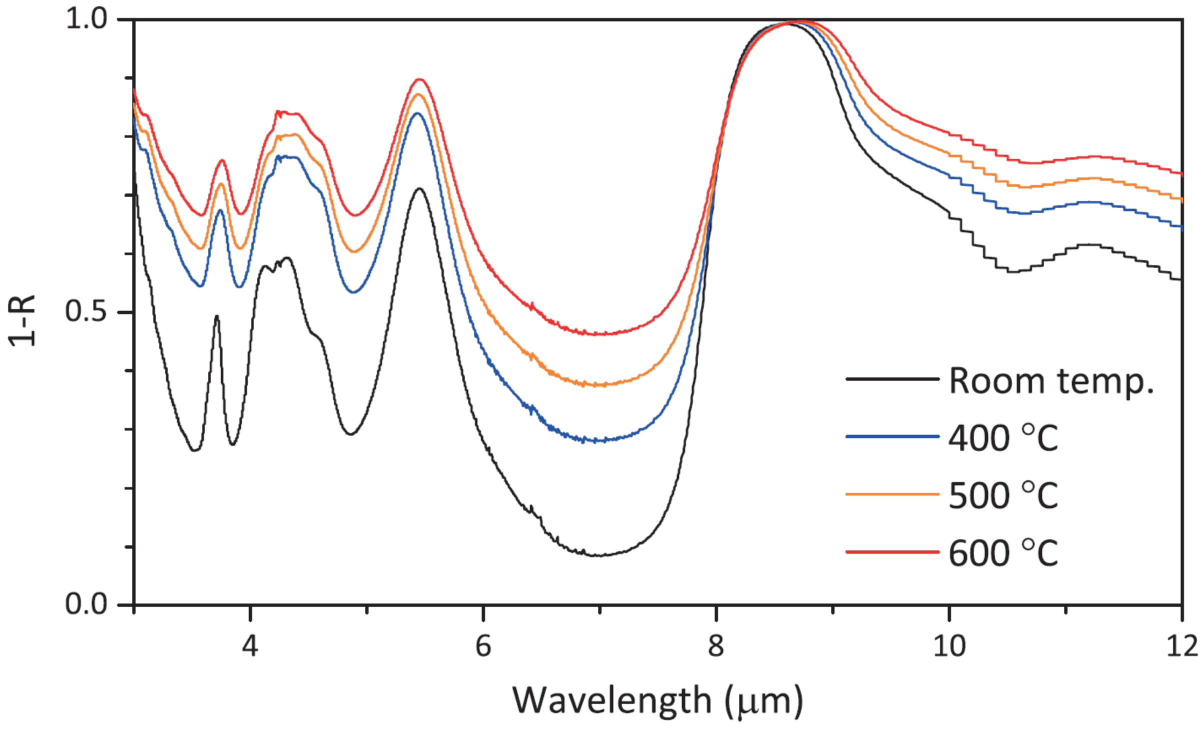

Figure S3: FTIR spectra for optimum device that is heated to different temperatures.
